# Indirect technique to estimate neonatal, infant, and under-five mortality rates for India and its states: A population-based cross-sectional study

**DOI:** 10.1371/journal.pone.0353613

**Published:** 2026-09-15

**Authors:** Mayank Singh, Anuj Singh, Abhay Kumar Tiwari

**Affiliations:** 1 Department of Epidemiology and Biostatistics, KAHER, Belgaum, Karnataka, India; 2 Department of Sciences (Mathematics), Indian Institute of Information Technology Ranchi, Ranchi, Jharkhand, India; 3 Department of Statistics, Institute of Science, Banaras Hindu University, Varanasi, Uttar Pradesh, India; All India Institute of Medical Sciences - Raipur, INDIA

## Abstract

**Background:**

Accurate assessment of child mortality rates is essential for policy formulation, resource allocation, and tracking progress toward Sustainable Development Goal-3 (SDG-3). The study of child mortality provides useful information to know the demographic situation of country. The death is vital event and recorded through the civil registration system. But in many developing countries, the quality of registered death is not very much reliable due to illiteracy and ignorance in population. So, the lack of accurate registration of death has forced demographers to explore the indirect techniques for estimating child mortality. In this paper, authors have utilized an indirect technique for estimating neonatal, infant and under-five mortality rate by using data on proportion of dead children and proportion of 4 + birth-order.

**Methods:**

The method is mainly based on technique of linear line regression analysis, where the proportion of dead infants among all children born to currently married females (15–49 years) and proportion of 4 + birth order are taken as the independent variables and the neonatal mortality rate, or infant mortality rate, or under-five mortality rate are used as the dependent variable. This study is based on data collected in fifth round of National Family Health Survey (NFHS), 2019−21. After applying the inclusion criteria, a total of 512,408 currently married women aged 15–49 years were included in the analysis.

**Result:**

For above mentioned child mortalities are calculated for India as well its major states. The actual and predicted mortality rates overlapped substantially when both predictors were used together, indicating the suitability of the proposed model. The combined-predictor models (proportion of dead children and proportion of 4 + birth-order) achieved higher R² values (0.91 for neonatal mortality rate (NMR), 0.92 for infant mortality rate (IMR), and 0.96 for under-five mortality rate (U5MR) along with the minor percentage differences between observed and estimated rates (mostly within ±10%) than single-predictor models.

**Conclusion:**

The findings suggest that integrating both the predictors, proportion of dead children and the proportion of higher birth order children improves the accuracy of indirect estimates of NMR, IMR, and U5MR. This approach may serve as a practical tool for generating timely mortality estimates in contexts where civil registration and survey data are incomplete or infrequent.

## Background

Neonatal, infant, and under-five mortality are critical indicators of a nation’s healthcare system, socioeconomic development, and overall well-being. In India, despite significant advancements in healthcare infrastructure and interventions, high mortality rates among newborns and young children persist, particularly in rural and marginalized communities. Accurate estimation of these mortality rates is essential for policy formulation, resource allocation, and monitoring progress towards Sustainable Development Goal 3 (SDG 3)—ensuring healthy lives and promoting well-being for all at all ages. Policy planners and health administrators rely on such data to design development strategies, address population health needs, and evaluate public health initiatives. But vital registration system often fails to capture all events due to limited administrative reach, illiteracy, and lack of awareness. While direct surveys is valuable for collecting detailed information but are expensive, time-consuming, and logistically challenging to conduct on a large scale. Given these limitations, indirect estimation methods offer a practical and cost-effective alternative by using readily available demographic, health, and socioeconomic indicators to infer mortality patterns and trends.

Demographers have been compelled to develop methods for predicting fundamental demographic parameters from erroneous or partial data due to the lack of reliable population counts and registration of important events. As we know, Brass focuses on methods for estimating mortality rates when data are limited or flawed [1]. His finding suggests techniques to derive reliable estimates of mortality even when data are incomplete or of poor quality. Another technique was used for estimating child mortality from information on proportion of dead children amongst children ever born to women with reference to the length of her marriage [2]. The alternate indirect techniques, such as those proposed for situations where mortality has been declining include the works of [3–5]. Islam and Alam used child mortality index (Ml) to calculate child mortality [6]. Neupert proposed a method to estimate infant mortality rates in small areas with limited data [7]. By using indirect estimation techniques based on fertility, childlessness, and total mortality data, it provides accurate estimates of infant mortality rates for various small geographic areas.

It is common knowledge that the percentage of dead among all children born to women in standard age groups can be used to estimate child mortality. It was first given by (Coal and Demeny, Brass and Coale) widely known as the Brass method [1,8]. On the other hand, the proportion of births of order four or higher (4 + birth order) has been consistently linked to elevated child mortality risks through mechanisms such as “resource dilution” where parental resources and attention are spread across more children, and “maternal depletion” where closely spaced and frequent pregnancies reduce maternal health reserves [9]. Higher birth order, defined as the fourth or subsequent birth within a family, has been associated with an increased risk of child mortality [10]. While some studies have reported a clear association between higher birth order and elevated mortality rates among children [11]. In 2003, Yadava and Tiwari applied an indirect estimation technique to calculate infant and under-five mortality rates using the proportion of dead children alone for India and Bangladesh [12]. Therefore, in this paper, we have evaluated the adequacy of this technique using recent NFHS data and extended it by incorporating the proportion of higher birth-order (4+) along with the proportion of dead children to improve estimation accuracy. The regression line analysis was conducted and tested using data from fifth round of NFHS.

## Materials and methods

### The data

The National Family Health Survey (NFHS), 2019–21 data-set has been used in this study. The NFHS is a large-scale, nationally representative survey conducted in India under the Ministry of Health and Family Welfare. NFHS-5 fieldwork for India was carried out by 17 Field Agencies in two phases: phase one from June 17, 2019, to January 30, 2020, and phase two from January 2, 2020, to April 30, 2021. Data were collected from 636,699 households, comprising 724,115 women and 101,839 males [13]. It provides comprehensive data on various health and demographic indicators, including fertility, maternal and child health, family planning, and nutrition at national, state and district level. By using information on the number of births and deaths within this period, along with the age at death of the deceased children, researchers may identify mortality rates. The present study used data from the Individual Recode (IR) file of NFHS-5 (2019–21). After applying the inclusion criteria, a total of 512,408 currently married women aged 15–49 years were included in the analysis. The information on proportion of dead children to currently married females (15–49 yrs.) and proportion of 4 + birth order may be calculated from the information given in the survey.

### The choice of predictors

In such estimation techniques, the choice of predictor (s) is very critical and important. The inappropriate choice may leads to misleading or not useful conclusion. The basic need in the choice of the variables(s) is that of significant correlation between the dependent and independent variable(s) and independent variable is easily obtainable. In selecting the predictor variables for the present study, we focused on indicators those are both theoretically and empirically robust in their association with child mortality. The information on proportion of dead children to currently married females 15–49 years is a well-established indirect indicator of child mortality and proportion of four or higher orders birth has been consistently linked to child mortality. Therefore, proportion of dead children to currently married females (15–49 years) have been identified from [12] and along with proportion of dead children we have also considered one more predictor variable, i.e., proportion of 4 + birth order for this study [9].

### Methodology

The essentially method is based on the technique of regression line. The regression line concept has been utilized to investigate the relationship between the dependent and independent variable(s).

The straight-line simple regression model with respect to population parameters β_0_ and β_1_ can be explained by:


$$\begin{array}{c} \text{Y }=\;\beta_{\text{0}}\;+\;\beta_{\text{1}}\text{ X} \end{array}$$

Where X and Y are independent and dependent variables respectively.

β_0_ indicates the average value of Y when X=0, and

β_1_ the slope of the line which indicates the change in the Y for per unit change in the X

For more than one independent variables, multiple regression analysis is used. The mathematical form of multiple regression model as:


$$\begin{array}{c} {\text{Y}}_{\text{i}}\;=\;\beta_{\text{0}}\;+\;\beta_{\text{1}}\;{\text{X}}_{\text{1i}}\;+\;\beta_{\text{2}}\;{\text{X}}_{\text{2i}}\;+\;\ldots \ldots +\;\beta_{\text{n}}\;{\text{X}}_{\text{ni}}\;+\;\epsilon_{\text{i}} \end{array}$$

Where:

Y_i_ is the ith value of dependent variable.

β_0_ is the intercept term, and

β_1,_ β_2,…._ β_n_ are regression coefficients representing the change in Y associated with a one-unit change in X_1,_ X_2,….,_ X_n_ respectively

ε_i_ is the error term

For the present study neonatal mortality rate (NMR)/ infant mortality rate (IMR)/ under five mortality rate (U5MR) was utilized as a dependent variable (Y). And the information on proportion of dead children among all children born to married mothers in the 15−49 age-group and proportion of 4 + birth orders considered as independent variable(s) namely X_1_ and X_2_ respectively [1,12]. The line of regression is drawn by taking major states of India using NFHS-5 data.

**Mathematical form of fitted equations**

**For Neonatal mortality rates (per thousand live births),**

Y = 458.41 X_1_ - 3.23, (R^2^ = 0.82) [X_1_ is the Proportion of dead children]

Y = 67.31 X_2_ + 10.28, (R^2^ = 0.80) [X_2_ is the Proportion of 4+ birth order]

Y = 267.66 X_1_ + 36.31 X_2_ + 0.875, (R^2^ = 0.91) [Both predictors X_1_ and X_2_ taken together]

**For Infant mortality rates (per thousand live births),**

Y = 599.23 X_1_ - 2.19, (R^2^ = 0.81) [X_1_ is the Proportion of dead children]

Y = 89.79 X_2_ + 15.13, (R^2^ = 0.83) [X_2_ is the Proportion of 4+ birth order]

Y = 325.62 X_1_ + 52.08 X_2_ + 3.68, (R^2^ = 0.92) [Both predictors X_1_ and X_2_ taken together]

**For Under-five mortality rates (per thousand live births),**

Y = 685.03 X_1_ + 0.386, (R^2^ = 0.86) [X_1_ is the Proportion of dead children]

Y = 100.53 X_2_ + 20.59, (R^2^ = 0.84) [X_2_ is the Proportion of 4+ birth order]

Y = 400.68 X_1_ + 54.13 X_2_ + 6.51, (R^2^ = 0.96) [Both predictors X_1_ and X_2_ taken together]

Thus, the proposed predictors (proportion of dead children along with the proportion of 4 + birth order) improve the value of R^2^ and provide better explanation of considered child mortalities estimates. The R² values indicate the proportion of variation in mortality explained by the regression models. Higher R² values represent better model fit, suggesting that the selected predictors account for a substantial proportion of the variability in mortality estimates.

### Model validation of the fitted model

1 **Cross Validation predication power**

A predictive model’s estimated performance in practice or the degree to which the suggested model is population-stabilized must be determined. In this regard, Herzberg’s cross validity prediction power (CVPP) technique has been applied [14], and it is described as [15]:


$$\begin{array}{c} \rho \text{2}\upsilon \;=\;\text{1}-\frac{\left({\text{n}}^{\text{2}}\;-\;\text{1})\;(\text{n }-\;\text{2})\;(\text{1},\;{\text{c}}^{\text{2}}\right)}{n(n-p-\text{1})\;(n-p-\text{2})} \end{array}$$

where n is the number of instances, c is the correlation coefficient between the estimated and observed values of the dependent variables, and p is the number of explanatory factors in the model.

2 **Shrinkage and Stability of R^2^**

The model’s shrinkage is indicated by the standard adjustment applied to the coefficient of determination to account for the arbitrary effects of further sampling:

$\text{Shrinkage }=\;|\;\rho^{\text{2}}\upsilon ,\;{\text{R}}^{\text{2}}|$

Where ρ^2^_υ_ is Cross Validation predication power (CVPP) and R^2^ is the coefficient of determination of the model. The stability of R^2^ of the model is equal to (1- Shrinkage) means lower shrinkage value provides more stability of the model.

**Note: All data management, data analysis, and statistical analyses were performed using Microsoft Excel and Stata Statistical Software, Version 16 (StataCorp LLC, College Station, TX, USA).**

## Results

Table 1 shows the proportion of dead children among all children born to married mothers in the 15–49 age groups and proportion of 4 + birth orders for India. Haryana, Gujarat, Maharashtra, Rajasthan, Punjab, Tamil Nadu, Karnataka, Orissa, West Bengal, Assam: these states exhibit lower mean numbers of children ever born compared to the national average. The Proportion of dead children is found to be high in four states, i.e., MP, UP, Bihar and Orissa. These states also have higher proportion of 4 + births.

**Table 1:** Proportion of dead children to currently married females in the age group 15-49 year and proportion of 4 + birth order for India and its some major states.

Region	Mean Children Ever Born	Mean Children alive	Prop of dead children	Proportion of 4 + birth order
India	1.75	1.65	0.057	0.198
Uttar Pradesh	1.95	1.80	0.077	0.329
Madhya Pradesh	1.92	1.77	0.078	0.243
Bihar	2.29	2.12	0.074	0.397
Haryana	1.73	1.63	0.058	0.163
Gujarat	1.71	1.62	0.053	0.177
Maharashtra	1.69	1.62	0.041	0.119
Rajasthan	1.80	1.71	0.050	0.228
Punjab	1.52	1.45	0.046	0.110
Tamil Nadu	1.51	1.45	0.040	0.055
Karnataka	1.61	1.54	0.043	0.114
Orissa	1.64	1.52	0.073	0.158
West Bengal	1.59	1.51	0.050	0.112
Assam	1.65	1.57	0.048	0.163

Table 2 indicates the estimates of neonatal mortality rate (NMR) for India and its major states. Results presented in the table shows that the estimated values of NMR for most of the states are closest to observed value of NMR. The neonatal mortality rate for India is estimated 23.0 by using proposed all three regression models, but there are wide variations found among the considered states. Three states located in central and eastern part of country like MP, UP and Bihar has relatively high neonatal mortality rates. India and its 13 major states under study the difference between observed and estimated value of NMR is less than 10% for 5 (India, Gujarat, Maharashtra, Rajasthan and Karnataka), 7 (India, UP, MP, Bihar, Haryana, Gujarat and Assam) and 10 (India, UP, MP, Bihar, Haryana, Gujarat, Maharashtra, Tamil Nadu, Karnataka and Orissa) for different predictors like proportion of dead children, proportion of 4 + birth order and both proportion of dead children and 4 + birth order taken together respectively. To evaluate the suitability of the suggested approach, a cut-off difference of 10% was set between the observed and estimated values.

**Table 2:** Estimated and observed Neonatal mortality rates (NMR) per 1000 live birth for India and its some major states.

Region	Observed NMR (Oi)	Estimated NMR (Ei) using Proportion of dead children (Yadava and Tiwari 2003)			Estimated NMR (Ei) using Proportion of 4 + birth order			Estimated NMR (Ei) using Proportion of dead children and Proportion of 4 + birth order		
		Prevalence	Confidence Interval at 95%	% difference between Oi and Ei	Prevalence	Confidence Interval at 95%	% difference between Oi and Ei	Prevalence	Confidence Interval at 95%	% difference between Oi and Ei
Uttar Pradesh	35.7	32.04	(29.5; 34.6)	10.26	32.43	(29.9; 35.0)	9.15	33.41	(30.8; 36.0)	6.41
Madhya Pradesh	28.9	32.59	(28.4; 36.7)	−12.88	26.65	(22.7; 30.6)	7.71	30.61	(26.5; 34.7)	−6.03
Bihar	34.4	30.80	(27.1; 34.5)	10.45	37.01	(33.1; 40.9)	−7.59	35.16	(31.3; 39.0)	−2.21
Haryana	21.1	23.27	(16.3; 30.3)	−10.24	21.26	(14.5; 28.0)	−0.71	22.27	(15.4; 29.2)	−5.48
Gujarat	21.3	20.90	(15.8; 26.0)	1.87	22.20	(16.9; 27.5)	−4.24	21.39	(16.2; 26.6)	−0.42
Maharashtra	16.6	15.76	(10.7; 20.8)	5.05	18.30	(13.0; 23.6)	−10.23	16.28	(11.2; 21.4)	1.91
Rajasthan	19.8	19.69	(15.6; 23.7)	0.53	25.64	(21.2; 30.1)	−29.47	22.54	(18.3; 26.8)	−13.83
Punjab	21.7	17.88	(11.7; 24.0)	17.58	17.69	(11.6; 23.8)	18.47	17.20	(11.1; 23.3)	20.75
Tamil Nadu	12.4	14.99	(9.3; 20.6)	−21.27	13.99	(8.5; 19.5)	−13.19	13.51	(8.1; 18.9)	−9.29
Karnataka	15.8	16.70	(11.0; 22.4)	−5.79	17.96	(12.1; 23.8)	−13.75	16.65	(10.9; 22.4)	−5.46
Orissa	26.7	30.32	(25.2; 35.5)	−13.37	20.92	(16.4; 25.5)	21.75	26.20	(21.3; 31.1)	2.03
West Bengal	15.4	19.84	(13.6; 26.1)	−29.16	17.83	(11.8; 23.8)	−16.06	18.41	(12.3; 24.5)	−19.86
Assam	22.2	19.00	(14.8; 23.2)	14.53	21.26	(16.9; 25.6)	4.36	19.77	(15.6; 24.0)	11.06
India	24.8	22.97	(22.0; 24.0)	7.38	23.62	(22.6; 24.6)	4.77	23.36	(22.3; 24.4)	5.80

Table 3 shows the estimates for infant mortality rate under the proposed predictors for India and its major states. To evaluate the suitability of the suggested approach, a cut-off difference of 10% was set between the observed and estimated values for the infant mortality rates. Out of 14 observed values for India and its states, there are 8 states (India, Bihar, Haryana, Gujarat, Maharashtra, Rajasthan, Punjab and Karnataka), 7 states (India, MP, Haryana, Gujarat, Maharashtra, Karnataka and Assam) and 11 states (India, Uttar Pradesh, Madhya Pradesh, Bihar, Haryana, Gujarat, Maharashtra, Rajasthan, Karnataka, Orissa and Assam) were found to be below 10% for different predictors, i.e., proportion of dead children, proportion of 4 + birth order and both proportion of dead children and 4 + birth order taken together respectively. Utilizing this standard, we noted the estimated values and observed values showed that they were fairly near to one another.

**Table 3:** Estimated and observed Infant mortality rates (IMR) per 1000 live birth for India and its some major states.

Region	Observed IMR	Estimated IMR using Proportion of dead children (Yadava and Tiwari 2003)			Estimated IMR using Proportion of 4 + birth order			Estimated IMR using Proportion of dead children and Proportion of 4 + birth order		
		Prevalence	Confidence Interval at 95%	% difference between Oi and Ei	Prevalence	Confidence Interval at 95%	% difference between Oi and Ei	Prevalence	Confidence Interval at 95%	% difference between Oi and Ei
Uttar Pradesh	49.70	43.90	(41.6; 46.2)	11.68	44.68	(42.4; 47.0)	10.10	45.87	(43.6; 48.2)	7.71
Madhya Pradesh	40.50	44.62	(41.1; 48.2)	−10.30	36.96	(33.5; 40.4)	8.63	41.78	(38.2; 45.3)	−3.29
Bihar	46.0	42.29	(38.9; 45.7)	8.07	50.79	(47.4; 54.2)	−10.40	48.54	(45.1; 52.0)	−5.51
Haryana	32.10	32.44	(26.4; 38.5)	−1.06	29.77	(23.8; 35.7)	7.25	31.00	(25.0; 37.0)	3.44
Gujarat	29.30	29.34	(24.6; 34.1)	−0.14	31.03	(26.2; 35.9)	−5.91	30.04	(25.2; 34.8)	−2.53
Maharashtra	23.60	22.62	(17.8; 27.4)	4.14	25.82	(20.8; 30.9)	−9.42	23.37	(28.0; 35.7)	0.97
Rajasthan	29.20	27.76	(24.0; 31.5)	4.92	35.61	(31.6; 39.6)	−21.95	31.84	(28.0; 35.7)	−9.05
Punjab	28.20	25.40	(19.3; 31.5)	9.94	25.01	(19.0; 31.1)	11.30	24.41	(18.4; 30.4)	13.44
Tamil Nadu	17.60	21.61	(16.5; 26.7)	−22.80	20.08	(15.1; 25.0)	−14.06	19.49	(14.6; 24.4)	−10.73
Karnataka	24.90	23.86	(18.9; 28.8)	4.19	25.37	(20.4; 30.4)	−1.90	23.78	(18.9; 28.7)	4.50
Orissa	35.08	41.65	(37.0; 46.3)	−18.72	29.32	(25.0; 33.6)	16.41	35.74	(31.2; 40.3)	−1.88
West Bengal	21.30	27.95	(21.9; 34.0)	−31.23	25.19	(19.3; 31.1)	−18.28	25.90	(20.0; 31.8)	−21.61
Assam	30.30	26.86	(22.9; 30.8)	11.37	29.77	(25.7; 33.9)	1.74	27.96	(23.9; 32.0)	7.71
India	34.60	32.04	(31.1; 33.0)	7.39	32.92	(32.0; 33.9)	4.87	32.61	(31.7; 33.5)	5.77

Table 4 reveals that on an average the estimated value of U5MR for most of the states and India are closer to observed value. Again, utilizing the cut off 10% difference, there are only 11 states are found to be within the limit (India, Uttar Pradesh, Madhya Pradesh, Bihar, Haryana, Gujarat, Maharashtra, Punjab, Tamil Nadu, Karnataka and Assam), 9 states (India, Uttar Pradesh, Madhya Pradesh, Bihar, Haryana, Gujarat, Tamil Nadu, West Bengal and Assam)and 13 considered states except Punjab for proportion of dead children, proportion of 4 + birth order and both proportion of dead children and 4 + birth order taken together respectively.

**Table 4:** Estimated and observed Under-five mortality rates (U5MR) per 1000 live birth for India and its some major states.

Region	Observed U5MR	Estimated U5MR using Proportion of dead children (Yadava and Tiwari 2003)			Estimated U5MR using Proportion of 4 + birth order			Estimated U5MR using Proportion of dead children and Proportion of 4 + birth order		
		Prevalence	Confidence Interval at 95%	% difference between Oi and Ei	Prevalence	Confidence Interval at 95%	% difference between Oi and Ei	Prevalence	Confidence Interval at 95%	% difference between Oi and Ei
Uttar Pradesh	57.5	53.08	(50.9; 55.2)	7.67	53.67	(51.5; 55.8)	6.65	55.13	(53.0; 57.3)	4.10
Madhya Pradesh	49.8	53.90	(50.7; 57.1)	−8.18	45.02	(41.9; 48.2)	9.65	50.96	(47.8; 54.1)	−2.26
Bihar	55.6	51.24	(48.2; 54.3)	7.87	60.51	(57.5; 63.5)	−8.78	57.74	(54.7; 60.8)	−3.80
Haryana	39.83	39.98	(34.3; 45.7)	−0.39	36.98	(31.3; 42.6)	7.16	38.49	(32.8; 44.2)	3.38
Gujarat	37.19	36.44	(31.9; 40.9)	2.01	38.39	(33.8; 42.9)	−3.22	37.17	(32.6; 41.7)	0.05
Maharashtra	26.95	28.76	(24.1; 33.4)	−6.72	32.56	(27.7; 37.4)	−20.80	29.54	(24.8; 34.2)	−9.61
Rajasthan	38.92	34.64	(31.1; 38.2)	11.00	43.51	(39.8; 47.2)	−11.80	38.88	(35.3; 42.5)	0.11
Punjab	35.29	31.93	(26.0; 37.9)	9.51	31.65	(25.7; 37.6)	10.31	30.91	(25.0; 36.8)	12.42
Tamil Nadu	25.48	27.61	(22.6; 32.6)	−8.35	26.12	(21.2; 31.1)	−2.52	25.40	(20.5; 30.3)	0.32
Karnataka	27.93	30.17	(25.4; 35.0)	−8.02	32.05	(27.2; 36.9)	−14.76	30.09	(25.3; 34.9)	−7.74
Orissa	43.61	50.51	(46.1; 54.9)	−15.82	36.48	(32.2; 40.7)	16.36	44.37	(40.0; 48.7)	−1.75
West Bengal	29.8	34.85	(29.0; 40.7)	−16.96	31.85	(26.1; 37.6)	−6.88	32.72	(26.9; 38.5)	−9.81
Assam	36.52	33.60	(29.7; 37.5)	7.99	36.98	(33.0; 41.0)	−1.26	34.75	(30.8; 38.7)	4.84
India	41.8	39.53	(38.6; 40.4)	5.43	40.50	(39.6; 41.4)	3.12	40.12	(39.2; 41.0)	4.03

The Table 2–4 also provides the 95% confidence interval for estimates of NMR, IMR and U5MR. For NMR, the observed values lie in the 95% confidence intervals for almost all states as well as India for proposed predictors, whereas the Yadava and Tiwari (2003) model and the higher-order birth alone as a predator miss the observed values in two and three states, respectively. For IMR, the regression model using both predictors demonstrate the strongest performance, with observed values consistently falling within its confidence limits and exhibiting smaller deviations from the true estimates. The proportion of dead-children model also performs reasonably well but shows notable mismatches in states such as Tamil-nadu and Orissa. The proportion of higher-order birth model proves less reliable, failing to capture observed values in approximately three states, including Bihar, Rajasthan, and West Bengal. For U5MR, the combined model yields the most consistent and accurate results, nearly always encompassing the observed values within its 95% confidence intervals and minimizing deviations. The proportion of dead-children model performs moderately well but with larger discrepancies in states such as Punjab and Orissa, while the higher-order birth model fails in around three states, notably Bihar, Rajasthan, and Maharashtra.

Table 5 presents regression models along with the coefficient of determination (R^2^), shrinkage, and stability of different mathematical models for estimation of neonatal mortality rate, infant mortality rate, and under-five mortality rate per 1,000 live births for India and some of its major states, based on data from the NFHS conducted between 2019−21. Among NMR, the stability values are 0.7652, 0.7442 and 0.8288 for first, second and third model respectively and shrinkage is minimum for third model. Similarly, among IMR, shrinkage is found to be 0.2454, 0.2239 and 0.1711 for 1^st^, 2^nd^ and 3^rd^ model respectively. And for U5MR, the stability is found to be lowest for combined model.

**Table 5:** Regression models, R^2^ (coefficient of determination), Shrinkage and Stability for India and its some major states.

Mathematical form	R^2^	Shrinkage |ρ^2^_υ_ - R^2^|	Stability (1- Shrinkage)
**For Neonatal Mortality Rate (per 1,000 live births)**			
1. Y = 458.41X_1_-3.23	0.82	0.2348	0.7652
2. Y = 67.31X_2_+10.28	0.80	0.2558	0.7442
3. Y = 267.66X_1_+36.31X_2_	0.91	0.1711	0.8288
**For Infant Mortality Rate (per 1,000 live births)**			
1. Y = 599.23X_1_-2.91	0.81	0.2454	0.7545
2. Y = 89.79X_2_+15.13	0.83	0.2239	0.7761
3. Y = 325.62X_1_+52.08X_2_ + 3.68	0.92	0.1533	0.8466
**Under Five Mortality Rate (per 1,000 live births)**			
1. Y = 685.03X_1_+0.386	0.86	0.1897	0.8103
2. Y = 100.53X_2_+20.59	0.84	0.2127	0.7872
3. Y = 400.68X_1_+54.13X_2_ + 6.51	0.96	0.0791	0.9209

*Footnote:* X_1_ = Proportion of dead children among all children born to currently married women aged 15–49 years.

X_2_ = Proportion of births of order 4 or higher (4+ birth order).

## Discussion

The present study considers the proportion of dead children among all children born to married women aged 15–49 years with the proportion of 4 + birth order children provide a more accurate and robust estimation of neonatal mortality rate (NMR), infant mortality rate (IMR), and under-five mortality rate (U5MR) in India than relying on either predictor alone. This approach advances earlier demographic research that highlighted the importance of multi-variable indirect estimation techniques in data-limited settings.

A notable strength of our approach is that indirect estimates were within ±10% of observed NFHS-5 values for most states, demonstrating practical utility where direct estimates are infeasible. This confirms the relevance of indirect methods with easily available data. Importantly, our analysis also illustrates how indirect methods can fill interim data gaps. The ability to generate reliable subnational estimates using readily available variables is particularly valuable for monitoring progress toward Sustainable Development Goal (SDG)- 3.

From a policy perspective, the proposed approach offers a rapid, low-cost means to estimate child mortality indicators in settings where CRVS systems remain weak. State and district health planners can use such interim estimates to identify high-burden regions, allocate resources for maternal and child health interventions, and monitor SDG 3 progress between NFHS rounds. At the global level, our approach shares conceptual similarities with the estimation framework employed by the UN Inter-agency Group for Child Mortality Estimation (UN IGME), which also relies on indirect indicators and survey-based measures to overcome data limitations [16]. However, unlike the UN IGME, which uses complex statistical modelling and Bayesian smoothing techniques to produce harmonised cross-country estimates, our model is specifically designed for subnational applications within India. It emphasizes methodological simplicity, transparency, and ease of replication at the state level, making it a practical tool for decentralized health planning. In this sense, it complements rather than replaces global estimation efforts. In summary, the study highlights the value of strengthening traditional indirect estimation methods by integrating multiple predictors, thereby enhancing both precision and applicability. The proposed model can serve as a cost-effective tool for policymakers in resource-limited settings, enabling evidence-based decision-making and more targeted child health interventions.

### Limitations

This study has certain limitations that should be acknowledged. First, the regression-based approach assumes a stable and consistent relationship between the selected predictors and child mortality outcomes across time and states, an assumption that may be affected by changes in healthcare access, fertility patterns, and socio-economic conditions. Second, while NFHS data provide nationally representative estimates, so there may be chance of occurrence of non-sampling and sampling errors, particularly in smaller states or subpopulations. Furthermore, while the regression residuals were generally small, they do suggest the presence of unexplained variation, which highlights the limitations.

## Conclusion

On the basis of the above results, it is reasonable to conclude that the information on proportion of dead children among all children born to married mothers in the 15–49 age-group and proportion of 4 + birth orders provide the better estimates of NMR, IMR and U5MR. The results also reveals that the estimates of these child mortalities are more closed to the observed values compare to estimates calculated using Yadava and Tiwari. While there is some shrinkage in the R^2^ values across the models, the stability remains relatively high for the model that when both predictors taken together, indicating robustness in its predictive ability. The percent difference between observed and estimated values of child mortalities by proposed regression models and positions of observed values in confidence interval indicate the accuracy of technique.

On the basis of above study, we may conclude that the proposed regression has the potential to provide quick and better estimates of neonatal, infant and under-five mortality from a limited and easily available data.

### Future research directions

Future studies could focus on validating the proposed indirect estimation method using alternative datasets, such as the Sample Registration System (SRS) or District Level Household and Facility Survey (DLHS), to test its robustness across different data sources and time periods. Incorporating additional predictors—such as maternal education, household wealth, and access to healthcare indicators etc. may improve the explanatory power and adaptability of the model’s. Moreover, longitudinal analyses could be used to examine how the relationship between predictors and mortality outcomes evolves over time, enabling periodic recalibration.

## Abbreviations

CRVS: Civil Registration and Vital Statistics
CVPP: Cross Validation Prediction Power
DHS: Demographic and Health Survey
DLHS: District Level Household and Facility Survey
IMR: Infant Mortality Rate
IIPS: International Institute for Population Sciences
MOHFW: Ministry of Health and Family Welfare
NFHS: National Family Health Survey
NMR: Neonatal Mortality Rate
Ml: Mortality index
SDG: Sustainable Development Goal
SRS: Sample Registration System
U5MR: Under-Five Mortality Rate
UN: United Nations
UN IGME: United Nations Inter-agency Group for Child Mortality Estimation
