# Supplementary material for: Indirect technique to estimate neonatal, infant, and under-five mortality rates for India and its states: A population-based cross-sectional study
Source: PLoS One. 2026 Sep 15;21(9):e0353613. doi: 10.1371/journal.pone.0353613 (PMC13577410; doi:10.1371/journal.pone.0353613)
Supplement: Supplementary file 1 [file pone.0353613.s003.docx]

**'Response to Reviewers’**

**Editor Comments**

**Comment-**1. It is highly recommended to check the manuscript for grammatical errors and proofread it thoroughly.

**Response:** We thank to editor for this recommendation. The manuscript has been thoroughly proofread to correct grammatical errors, improve sentence structure, and enhance overall readability.
**Comment-**2. Please, remove irrelevant sentences from the introduction part of the abstract.

**Response:** We appreciate the editor’s suggestion. The introduction portion of the abstract has been revised to remove irrelevant sentences and ensure it is concise, focused, and directly aligned with the study objectives.

**Comment-**3. It is challenging to follow the manuscript. There is no proper sequence between the sentences within the paragraph. Please correct it to make it easily readable.

**Response:** We thank the editor for this valuable feedback. The whole manuscript has been thoroughly revised to improve logical flow and coherence within paragraphs. Sentences have been reordered where necessary to maintain a clear sequence of ideas, and transitions have been added to enhance readability and overall narrative structure.

**Comment-**4. “But some of the data sources in India are…”. What is the relevance of this statement? This appears to be somewhat controversial, as the analysis in this study is based on secondary data.

**Response:** We thank the editor for pointing out this concern. The sentence in question has been reworded to clarify its intent and avoid any potential misunderstanding. The purpose was not to undermine the NFHS dataset used in our analysis, but rather to acknowledge the broader context that certain data sources in India, such as the civil registration system, have incomplete coverage, especially in rural areas.

**Comment-**5. Which DHS data was used in the manuscript?

**Response:** We thank to editor for requesting this clarification. The analysis in this manuscript is based on the National Family Health Survey (NFHS-5, 2019–21) dataset (which is India’s version of the Demographic and Health Survey (DHS) program-7).

**Comment-**6. References are not in the Vancouver style.

**Response:** Thank you for your valuable comment. We have carefully revised and reformatted all references according to the Vancouver style, adhering to the PLOS ONE journal guidelines.

**Comment-**7. Some factors which affect the mortality rates were not considered in this study. Mention the limitations regarding the residuals.

**Response:** We thank the reviewer for this valuable observation. We acknowledge that several socioeconomic and healthcare-related factors influencing child mortality could not be incorporated due to data availability constraints. Furthermore, while the regression residuals were generally small, they do suggest the presence of unexplained variation, which highlights the limitations.

**Comment-**8. Tables 2 and 3: Mention the 95% CI for the proportion.

**Response:** We appreciate the reviewer’s suggestion. We have implemented the 95% confidence interval (CI) for the proportions from each models in the revised manuscript.

**Comment-**9. Table 5: What are X₁ and X₂? Please mention in the footnotes.

**Response:** We thank the reviewer for pointing this out. We have now added a footnote below Table 5 clarifying the definitions of X_1_ and X_2_ as follows:

- X_1_ = Proportion of dead children among all children born to currently married women aged 15–49 years.
- X_2_ = Proportion of births of order 4 or higher (4+ birth order).

**Review Comments to the Author**
**Reviewer #1:** This study presents an indirect technique for estimating child mortality rates in India using regression-based models. The approach is valuable given the limitations of direct surveys and civil registration systems. However, the manuscript requires improvements in clarity, methodological transparency, and discussion of limitations.

**Comment-1:** The abstract lacks statistical details on the results. Including R² values or error margins would make it more informative.

**Response:** We thank the reviewer for this observation. The abstract has been revised to include key statistical measures from our analysis in the revised manuscript.

**Comment**-**2:** The introduction highlights the limitations of existing data sources but does not clearly justify why the selected variables (proportion of dead children and 4+ birth order) were chosen over other possible indicators. Please provide a stronger rationale.

**Response:** We thank the reviewer for this valuable suggestion. In the revised manuscript, we have strengthened the rationale for selecting the two predictor variables. Specifically, we now provide both a theoretical and empirical justification for using the proportion of dead children ever born and proportion of higher-order births (4+).

Furthermore, preliminary correlation analysis using NFHS-5 data demonstrates that both predictors are strongly associated (Pearson’s r > 0.80) with neonatal, infant, and under-five mortality rates, outperforming alternative socioeconomic or healthcare access variables.

**Comment-3:** The regression analysis approach should be more explicitly described.

**Response:** We appreciate the reviewer’s suggestion. The Methodology section has been substantially revised to provide a clearer and more detailed description of the regression analysis.

**Comment-4:** It is unclear how the regression models were validated. Were any statistical goodness-of-fit measures, such as R² or RMSE, used?

**Response:** We thank the reviewer for this observation. The revised Methodology and Model Validation sections now provide a detailed explanation of the model validation process. Goodness-of-fit was assessed using the coefficient of determination (R^2^) for each model. Predictive stability was evaluated using Herzberg’s Cross Validity Prediction Power (CVPP) and shrinkage, with smaller shrinkage values indicating greater stability. Practical predictive accuracy was measured by comparing estimated mortality rates with observed NFHS-5 values for each state, applying a ±10% deviation criterion for acceptability.

**Comment-5:** The manuscript should clarify whether any adjustments (e.g., for confounding variables or biases) were made in the models.

**Response:** We thank the reviewer for raising this important point. In Methodology section, it explicitly states that no additional adjustments for potential confounding variables or biases were applied beyond the two selected predictors (proportion of dead children and proportion of 4+ birth orders). This choice was deliberate to preserve the simplicity and replicability of the indirect estimation approach, particularly in data-limited contexts.

**Comment-6:** The manuscript states that data from the National Family Health Survey (2019-21) were used. Were there any data exclusion criteria? How were missing values handled?

**Response:** We thank the reviewer for this important query. The analysis was conducted using the publicly available data from the National Family Health Survey (2019–21). No exclusion criteria were applied beyond what is already embedded in the NFHS sampling design. With respect to missing values, the dataset used contains very few instances of missing or inconsistent responses for the variables of interest. These cases were automatically excluded from the analysis through listwise deletion, which had negligible impact on the overall sample size and results.

**Comment-7:** The results indicate a strong alignment between actual and expected rates, but additional statistical evidence (e.g., confidence intervals, p-values) would enhance the credibility of the findings.

**Response:** We thank the reviewer for this thoughtful suggestion. In the revised manuscript, we have incorporated 95% confidence intervals (CIs) for the proportions obtained from each model to provide stronger statistical support for the findings. In addition, accuracy was assessed by comparing the estimated mortality rates with the observed NFHS-5 values for each state, using a ±10% deviation criterion as the threshold for acceptability.

**Comment-8:** More comparative analysis with other indirect estimation methods (e.g., Brass method) would strengthen the argument.

**Response:** We thank the reviewer for this valuable suggestion. In the present study, we have compared our proposed model with the method of Yadava and Tiwari (2003): “An indirect technique for estimations of infant and child mortality: Data analysis from India and Bangladesh” (*Health and Population Perspectives and Issues*, 26(2), 67–73). This method is conceptually based on the Brass indirect estimation framework, as it employs proportions of dead children to indirectly estimate infant and child mortality in settings with incomplete vital registration. Thus, the comparison already reflects a Brass-derived approach that has been widely applied in the Indian and Bangladeshi context, making it a relevant benchmark for evaluating our model.

**Comment-9:** The manuscript should discuss the potential policy implications of using this indirect method. A brief comparison with other similar global approaches (e.g., UN Inter-agency Group for Child Mortality Estimation) would be beneficial.

**Response:** We thank the reviewer for this valuable suggestion. In the revised manuscript, we included in the Discussion section to explicitly address the potential policy implications of the proposed indirect method, particularly for subnational health planning in contexts with incomplete civil registration systems. We have also added a brief comparison with the approach used by the UN Inter-agency Group for Child Mortality Estimation (UN IGME), highlighting similarities in the use of indirect indicators and differences in modeling complexity, scope, and intended application.

**Comment-10:** The manuscript should explicitly acknowledge limitations, such as assumptions in the regression model, potential biases in NFHS data, and the exclusion of certain socioeconomic factors.

**Response:** We thank the reviewer for this insightful comment. In the revised manuscript, we have added a dedicated section on “Limitations,” where we discuss assumptions inherent in the regression model, potential biases in NFHS data, and the non-inclusion of certain socioeconomic and healthcare-related factors due to data availability constraints.

**Comment-11:** Future research directions should be outlined, including how this method could be improved or validated with other datasets.

**Response:** We thank the reviewer for this suggestion. In the revised manuscript, we have added a “Future Research Directions” paragraph. This addition outlines potential next steps, including validating the proposed method using alternative datasets such as the Sample Registration System (SRS) and District Level Household and Facility Survey (DLHS), incorporating additional socioeconomic and health system predictors, applying longitudinal analyses for model recalibration, and exploring state-specific adjustments to improve accuracy.

**Comment-12:** Some sentences are unclear or grammatically incorrect. For example:
“But some of the data-source in India is not dependable like civil registration system…” → Consider rewording for clarity:

“Some data sources in India, such as the civil registration system, are unreliable due to incomplete coverage, particularly in rural areas.”

**Response:** We thank the reviewer for this valuable observation. The sentence has been reworded for clarity and improved grammar in the revised manuscript.

**Comment-13:** - Proofreading is needed to improve readability.

**Response:** We appreciate the reviewer’s observation. The entire manuscript has undergone thorough proofreading to improve readability, correct grammatical errors, and enhance clarity in the revised manuscript.

**Reviewer #2:** Thank you for preparing this important manuscript. I am really unable to appropriately assess the technical parts of this paper because the written English in this manuscript needs significant improvement. I know that English is not the first language of the authors and commend them in putting together this paper; however, in its current form it is very confusing and difficult to understand the writing. I think this paper needs to be edited by someone who speaks fluent English and then resubmitted for review.
There is also no discussion section of the paper which needs to be added.
In addition: there is no such method as "regression line analysis", I am assuming the authors meant a linear regression-if so this needs to be corrected in the entire paper.

**Response:** We thank the reviewer for these constructive suggestions. In the revised manuscript, we have added a comprehensive Discussion section that interprets our findings in the context of previous studies, outlines policy implications, and addresses limitations.

Regarding the term *“regression line analysis”*, we agree that this is not standard terminology. Throughout the manuscript, including the Methodology section, we have replaced *“regression line analysis”* with the appropriate statistical terms “*linear line regression technique” (*general, covers both simple and multiple regression*)”*.
