# Supplementary material for: Indirect technique to estimate neonatal, infant, and under-five mortality rates for India and its states: A population-based cross-sectional study
Source: PLoS One. 2026 Sep 15;21(9):e0353613. doi: 10.1371/journal.pone.0353613 (PMC13577410; doi:10.1371/journal.pone.0353613)
Supplement: Supplementary file 2 [file pone.0353613.s004.docx]

The Editor
PLOS ONE Journal
11 June 2026

**Subject: Reviewer Comments on**
*“Indirect Technique to Estimate Neonatal, Infant, and Under-Five Mortality Rates for India and its States”*
(Manuscript ID: PONE-D-25-04899R1)

Dear Editor,

I appreciate the authors’ efforts in addressing an important and relevant topic. The manuscript provides useful insights into the *Indirect Technique to Estimate Neonatal, Infant, and Under-Five Mortality Rates for India and its States*. However, a few minor revisions are required to improve clarity and methodological transparency.

**Reviewer Comments**

**1. Title:**
Please consider including the study design in the title.

**2. Abstract:**
2.1 It is recommended to add keywords as a subheading.
2.2 In the Methods section, please specify the sample size used in the analysis, including the number of currently married women aged 15–49 years included from the National Family Health Survey (NFHS-5, 2019–21).

**3. Methodology (Page 6):**
Please provide appropriate references supporting the regression-based mortality estimation method and justify the rationale for selecting the predictor variables used in the analysis.

**4. Software:**
Please specify the statistical software used, along with its version.

**5. Formatting requirement:**
Please include the following section before the References, in accordance with journal guidelines:

- List of Abbreviations (in alphabetical order)

**Recommendation**

Minor Revision

Best regards,
**Zainab Mahmood Al-Zadjali**

Reviewer
Department of Family Medicine and Public Health
College of Medicine and Health Sciences
Sultan Qaboos University
Muscat, Sultanate of Oman

zainab.alzadjali@yahoo.com
