# Supplementary material for: Indirect technique to estimate neonatal, infant, and under-five mortality rates for India and its states: A population-based cross-sectional study
Source: PLoS One. 2026 Sep 15;21(9):e0353613. doi: 10.1371/journal.pone.0353613 (PMC13577410; doi:10.1371/journal.pone.0353613)
Supplement: Supplementary file 3 [file pone.0353613.s005.docx]

**'Response to Reviewers’**

**Reviewer Comments**

**Title:**

**Comment 1:** Please consider including the study design in the title.

*Reply:* Thank you for this valuable suggestion. We agree that including the study design in the title improves clarity and better reflects the methodological framework of the study. Accordingly, we have revised the title to include the study design as suggested.

**Abstract:**

**Comment 2.1:** It is recommended to add keywords as a subheading.

*Reply:* Thank you for the helpful suggestion. We agree that including keywords as a separate subheading after the abstract improves the discoverability and indexing of the manuscript. Accordingly, we have added a Keywords section below the abstract in the revised manuscript.

**Comment** **2.2:** In the Methods section, please specify the sample size used in the analysis, including the number of currently married women aged 15–49 years included from the National Family Health Survey (NFHS-5, 2019–21).

*Reply:* Thank you for this valuable suggestion. We agree that explicitly reporting the sample size improves the methodological clarity of the study. Accordingly, we have revised the Methods section to specify the total number of participants included in the analysis, namely the number of currently married women aged 15–49 years selected from the National Family Health Survey (NFHS-5, 2019–21).

**Methodology (Page 6):**

**Comment** **3:** Please provide appropriate references supporting the regression-based mortality estimation method and justify the rationale for selecting the predictor variables used in the analysis.

*Reply****:*** The regression-based indirect estimation method used in this study is supported by established demographic literature, including Brass (1975) [Reference 1], which provides the theoretical foundation for indirect mortality estimation using incomplete data, and Yadava and Tiwari (2003) [Reference 12], who successfully applied regression-based techniques for estimating infant and child mortality in India and Bangladesh.

The predictor variables were selected based on both theoretical relevance and empirical evidence. The proportion of dead children among all children born to currently married women aged 15–49 years is a widely accepted indirect indicator of child mortality and has been used in previous demographic estimation studies. In addition, the proportion of births of order 4 or higher (4+ birth order) was included because higher birth order is associated with elevated child mortality risks due to maternal depletion and resource dilution. These predictors also showed strong associations with neonatal, infant, and under-five mortality rates in the present analysis.

**Software:**

**Comment** **4:** Please specify the statistical software used, along with its version.

*Reply:* Thank you for this valuable suggestion. We have now specified the software used in the revised manuscript. All data management, data analysis, and statistical analyses were performed using Microsoft Excel and Stata Statistical Software, Version 16 (StataCorp LLC, College Station, TX, USA).

**Formatting requirement:**

**Comment** **5:** Please include the following section before the References, in accordance with journal guidelines:

- List of Abbreviations (in alphabetical order)

*Reply:* Thank you for this helpful suggestion. In accordance with the journal guidelines, we have added a List of Abbreviations section before the References and arranged all abbreviations in alphabetical order.
