# Supplementary material for: Indirect technique to estimate neonatal, infant, and under-five mortality rates for India and its states: A population-based cross-sectional study
Source: PLoS One. 2026 Sep 15;21(9):e0353613. doi: 10.1371/journal.pone.0353613 (PMC13577410; doi:10.1371/journal.pone.0353613)
Supplement: Supplementary file 4 [file pone.0353613.s006.docx]

29 June 2026

**The Editor**
**PLOS ONE**

**Subject: Reviewer Comments on "Indirect Technique to Estimate Neonatal, Infant, and Under-Five Mortality Rates for India and its States: A Population-Based Cross-Sectional Study" (Manuscript ID: PONE-D-25-04899R2)**

Dear Editor,

I appreciate the authors' efforts in addressing an important and relevant public health topic. The manuscript provides useful insights into the indirect estimation of neonatal, infant, and under-five mortality rates in India and its states using a population-based cross-sectional approach.

After reviewing the revised manuscript and the authors' responses, I am satisfied that the authors have adequately addressed all reviewer comments and concerns raised during the previous review rounds. The revisions have improved the clarity and quality of the manuscript.

**Reviewer Comments**

**1. Title**
The authors have addressed the comment regarding the inclusion of the study design in the title. However, I noticed a punctuation/typographical error, with a duplicated colon before “A Population-Based Cross-Sectional Study.”

**2. Abstract**
The authors have adequately addressed the comments regarding the abstract, except for the inclusion of the sample size in the abstract.

**3. Methodology (Page 6)**
The authors have provided appropriate references supporting the regression-based mortality estimation method and have justified the rationale for selecting the predictor variables used in the analysis.

**4. Software**
The authors have specified the statistical software used, including the software version.

**5. Formatting Requirements**
The authors have addressed the formatting requirements in accordance with the journal guidelines and added an alphabetical list of abbreviations.

**Recommendation**

**Accept.**

Best regards,

**Zainab Mahmood Al-Zadjali**

Reviewer
Department of Family Medicine and Public Health
College of Medicine and Health Sciences
Sultan Qaboos University
Muscat, Sultanate of Oman
[zainab.alzadjali@yahoo.com](mailto:zainab.alzadjali@yahoo.com)
