# Supplementary material for: Indirect technique to estimate neonatal, infant, and under-five mortality rates for India and its states: A population-based cross-sectional study
Source: PLoS One. 2026 Sep 15;21(9):e0353613. doi: 10.1371/journal.pone.0353613 (PMC13577410; doi:10.1371/journal.pone.0353613)
Supplement: Supplementary file 5 [file pone.0353613.s007.docx]

**'Response to Reviewers & Editor’**

We sincerely thank the Academic Editor for the careful evaluation of our manuscript and for providing us with the opportunity to revise it. We are grateful for the valuable comments and suggestions, which have helped us improve the quality and clarity of the manuscript.

**Journal Requirements:**

**1.** If the reviewer comments include a recommendation to cite specific previously published works, please review and evaluate these publications to determine whether they are relevant and should be cited. There is no requirement to cite these works unless the editor has indicated otherwise.

*Reply:* We carefully reviewed the reviewers' comments and confirmed that no specific previously published works were recommended for citation. Therefore, no additional references were added on this basis.

**2.** Please review your reference list to ensure that it is complete and correct. If you have cited papers that have been retracted, please include the rationale for doing so in the manuscript text, or remove these references and replace them with relevant current references. Any changes to the reference list should be mentioned in the rebuttal letter that accompanies your revised manuscript. If you need to cite a retracted article, indicate the article’s retracted status in the References list and also include a citation and full reference for the retraction notice.

*Reply:* We carefully reviewed the reference list to ensure that it is complete and accurate. We also verified that none of the cited references have been retracted. Accordingly, no changes related to retracted publications were required.

**Reviewers Comment**

**Reviewer-3**

**1. Title**

**Comment 1:** The authors have addressed the comment regarding the inclusion of the study design in the title. However, I noticed a punctuation/typographical error, with a duplicated colon before “A Population-Based Cross-Sectional Study.

*Reply:* Thank you for your observation. We have corrected the punctuation/typographical error by removing the duplicated colon from the title. The title has been revised accordingly.

**2. Abstract**

**Comment 2:** The authors have adequately addressed the comments regarding the abstract, except for the inclusion of the sample size in the abstract.

*Reply:* Thank you for your valuable comment. We have added the study sample size in the Abstract, as suggested. The revised Abstract now includes this information.

**3. Methodology (Page 6)**

**Comment** **3:** The authors have provided appropriate references supporting the regression-based mortality estimation method and have justified the rationale for selecting the predictor variables used in the analysis.

*Reply:* We sincerely thank the reviewer for the positive assessment.

**4. Software**

**Comment** **4:** The authors have specified the statistical software used, including the software version.

*Reply****:*** We thank the reviewer for this positive comment.

**5. Formatting Requirements**

**Comment** **5:** The authors have addressed the formatting requirements in accordance with the journal guidelines and added an alphabetical list of abbreviations.

*Reply****:*** We thank the reviewer for this positive comment.

**Reviewer-4**

This interesting manuscript has been substantially improved and the authors have addressed most of the comments from the previous reviews. I have a few remaining comments:

**Comment** **1:** Though the readability is markedly improved, there are a few remaining grammatical errors - I suggest an additional round of proofreading by a fluent English speaker.

*Reply****:*** We sincerely thank the reviewer for this helpful suggestion. The manuscript has undergone an additional round of careful English proofreading, and the remaining grammatical and typographical errors have been corrected throughout the manuscript.

**Comment** **2:** R-squared values are provided for all models and offer meaningful information regarding model fit. I suggest adding a sentence regarding interpretation of R-squared for the benefit of readers with limited statistical training.

*Reply****:*** Thank you for your valuable suggestion. We have added a brief sentence explaining the interpretation of the R-squared values to help readers better understand the model fit. The manuscript has been revised accordingly.
