# Supplementary material for: Indirect technique to estimate neonatal, infant, and under-five mortality rates for India and its states: A population-based cross-sectional study
Source: PLoS One. 2026 Sep 15;21(9):e0353613. doi: 10.1371/journal.pone.0353613 (PMC13577410; doi:10.1371/journal.pone.0353613)
Supplement: Peer review history

# Peer review: Decision Letter 0

**DOI:** 10.1371/journal.pone.0353613.r001

**Authors:** Ashish Wasudeo Khobragade

10 Aug 2025

Dear Dr. Singh,

plosone@plos.org. When you're ready to submit your revision, log on to https://www.editorialmanager.com/pone/ and select the 'Submissions Needing Revision' folder to locate your manuscript file.

- A rebuttal letter that responds to each point raised by the academic editor and reviewer(s). You should upload this letter as a separate file labeled 'Response to Reviewers'.
- A marked-up copy of your manuscript that highlights changes made to the original version. You should upload this as a separate file labeled 'Revised Manuscript with Track Changes'.
- An unmarked version of your revised paper without tracked changes. You should upload this as a separate file labeled 'Manuscript'.

If you would like to make changes to your financial disclosure, please include your updated statement in your cover letter. Guidelines for resubmitting your figure files are available below the reviewer comments at the end of this letter.

If applicable, we recommend that you deposit your laboratory protocols in protocols.io to enhance the reproducibility of your results. Protocols.io assigns your protocol its own identifier (DOI) so that it can be cited independently in the future. For instructions see: https://journals.plos.org/plosone/s/submission-guidelines#loc-laboratory-protocols. Additionally, PLOS ONE offers an option for publishing peer-reviewed Lab Protocol articles, which describe protocols hosted on protocols.io. Read more information on sharing protocols at https://plos.org/protocols?utm_medium=editorial-email&utm_source=authorletters&utm_campaign=protocols.

We look forward to receiving your revised manuscript.

Kind regards,

Ashish Wasudeo Khobragade, MD

Academic Editor

PLOS ONE

Journal requirements:

When submitting your revision, we need you to address these additional requirements.

1. Please ensure that your manuscript meets PLOS ONE's style requirements, including those for file naming. The PLOS ONE style templates can be found at

https://journals.plos.org/plosone/s/file?id=wjVg/PLOSOne_formatting_sample_main_body.pdf and

https://journals.plos.org/plosone/s/file?id=ba62/PLOSOne_formatting_sample_title_authors_affiliations.pdf

2. When completing the data availability statement of the submission form, you indicated that you will make your data available on acceptance. We strongly recommend all authors decide on a data sharing plan before acceptance, as the process can be lengthy and hold up publication timelines. Please note that, though access restrictions are acceptable now, your entire data will need to be made freely accessible if your manuscript is accepted for publication. This policy applies to all data except where public deposition would breach compliance with the protocol approved by your research ethics board. If you are unable to adhere to our open data policy, please kindly revise your statement to explain your reasoning and we will seek the editor's input on an exemption. Please be assured that, once you have provided your new statement, the assessment of your exemption will not hold up the peer review process.

3. PLOS requires an ORCID iD for the corresponding author in Editorial Manager on papers submitted after December 6th, 2016. Please ensure that you have an ORCID iD and that it is validated in Editorial Manager. To do this, go to ‘Update my Information’ (in the upper left-hand corner of the main menu), and click on the Fetch/Validate link next to the ORCID field. This will take you to the ORCID site and allow you to create a new iD or authenticate a pre-existing iD in Editorial Manager.

If the reviewer comments include a recommendation to cite specific previously published works, please review and evaluate these publications to determine whether they are relevant and should be cited. There is no requirement to cite these works unless the editor has indicated otherwise.

Additional Editor Comments:

1. It is highly recommended to check the manuscript for grammatical errors and proofread it thoroughly.

2. Please, remove irrelevant sentences from the introduction part of the abstract.

3. It is challenging to follow the manuscript. There is no proper sequence between the sentences within the paragraph. Please correct it to make it easily readable.

4. “But some of the data sources in India are…”. What is the relevance of this statement? This appears to be somewhat controversial, as the analysis in this study is based on secondary data.

5. Which DHS data was used in the manuscript?

6. References are not in the Vancouver style.

7. Some factors which affect the mortality rates were not considered in this study. Mention the limitations regarding the residuals.

8. Tables 2 and 3: Mention the 95% CI for the proportion.

9. Table 5: What are X₁ and X₂? Please mention in the footnotes.

Comments from Journal Office:

We suggest you have a fluent English-language speaker thoroughly copyedit your manuscript for language usage, spelling, and grammar. If you do not know anyone who can do this, you may wish to consider employing a professional scientific editing service.

[Note: HTML markup is below. Please do not edit.]

Reviewers' comments:

Reviewer's Responses to Questions

**Comments to the Author**

1. Is the manuscript technically sound, and do the data support the conclusions?

Reviewer #1: Partly

Reviewer #2: Partly

**********

2. Has the statistical analysis been performed appropriately and rigorously?

Reviewer #1: No

Reviewer #2: I Don't Know

**********

3. Have the authors made all data underlying the findings in their manuscript fully available?

The PLOS Data policy

Reviewer #1: No

Reviewer #2: Yes

**********

4. Is the manuscript presented in an intelligible fashion and written in standard English?

Reviewer #1: No

Reviewer #2: No

**********

Reviewer #1: This study presents an indirect technique for estimating child mortality rates in India using regression-based models. The approach is valuable given the limitations of direct surveys and civil registration systems. However, the manuscript requires improvements in clarity, methodological transparency, and discussion of limitations.

- The abstract lacks statistical details on the results. Including R² values or error margins would make it more informative.

- The introduction highlights the limitations of existing data sources but does not clearly justify why the selected variables (proportion of dead children and 4+ birth order) were chosen over other possible indicators. Please provide a stronger rationale.

- The regression analysis approach should be more explicitly described.

- It is unclear how the regression models were validated. Were any statistical goodness-of-fit measures, such as R² or RMSE, used?

- The manuscript should clarify whether any adjustments (e.g., for confounding variables or biases) were made in the models.

- The manuscript states that data from the National Family Health Survey (2019-21) were used. Were there any data exclusion criteria? How were missing values handled?

- The results indicate a strong alignment between actual and expected rates, but additional statistical evidence (e.g., confidence intervals, p-values) would enhance the credibility of the findings.

- More comparative analysis with other indirect estimation methods (e.g., Brass method) would strengthen the argument.

- The manuscript should discuss the potential policy implications of using this indirect method.

A brief comparison with other similar global approaches (e.g., UN Inter-agency Group for Child Mortality Estimation) would be beneficial.

- The manuscript should explicitly acknowledge limitations, such as assumptions in the regression model, potential biases in NFHS data, and the exclusion of certain socioeconomic factors.

- Future research directions should be outlined, including how this method could be improved or validated with other datasets.

- Some sentences are unclear or grammatically incorrect. For example:

“But some of the data-source in India is not dependable like civil registration system…” → Consider rewording for clarity:

“Some data sources in India, such as the civil registration system, are unreliable due to incomplete coverage, particularly in rural areas.”

- Proofreading is needed to improve readability.

Reviewer #2: Thank you for preparing this important manuscript. I am really unable to appropriately assess the technical parts of this paper because the written English in this manuscript needs significant improvement. I know that English is not the first language of the authors and commend them in putting together this paper; however, in its current form it is very confusing and difficult to understand the writing. I think this paper needs to be edited by someone who speaks fluent English and then resubmitted for review.

There is also no discussion section of the paper which needs to be added.

In addition: there is no such method as "regression line analysis", I am assuming the authors meant a linear regression-if so this needs to be corrected in the entire paper.

Thank you for the opportunity to review this paper.

**********

what does this mean?). If published, this will include your full peer review and any attached files.

If you choose “no”, your identity will remain anonymous but your review may still be made public.

**Do you want your identity to be public for this peer review?**  For information about this choice, including consent withdrawal, please see our Privacy Policy

Reviewer #1: No

Reviewer #2: No

**********

[NOTE: If reviewer comments were submitted as an attachment file, they will be attached to this email and accessible via the submission site. Please log into your account, locate the manuscript record, and check for the action link "View Attachments". If this link does not appear, there are no attachment files.]

While revising your submission, please upload your figure files to the Preflight Analysis and Conversion Engine (PACE) digital diagnostic tool, https://pacev2.apexcovantage.com/. PACE helps ensure that figures meet PLOS requirements. To use PACE, you must first register as a user. Registration is free. Then, login and navigate to the UPLOAD tab, where you will find detailed instructions on how to use the tool. If you encounter any issues or have any questions when using PACE, please email PLOS at figures@plos.org. Please note that Supporting Information files do not need this step.

# Author comment: Author response to Decision Letter 1

**DOI:** 10.1371/journal.pone.0353613.r002

19 Sep 2025

'Response to Reviewers’

Editor Comments

Comment-1. It is highly recommended to check the manuscript for grammatical errors and proofread it thoroughly.

Response: We thank to editor for this recommendation. The manuscript has been thoroughly proofread to correct grammatical errors, improve sentence structure, and enhance overall readability.

Comment-2. Please, remove irrelevant sentences from the introduction part of the abstract.

Response: We appreciate the editor’s suggestion. The introduction portion of the abstract has been revised to remove irrelevant sentences and ensure it is concise, focused, and directly aligned with the study objectives.

Comment-3. It is challenging to follow the manuscript. There is no proper sequence between the sentences within the paragraph. Please correct it to make it easily readable.

Response: We thank the editor for this valuable feedback. The whole manuscript has been thoroughly revised to improve logical flow and coherence within paragraphs. Sentences have been reordered where necessary to maintain a clear sequence of ideas, and transitions have been added to enhance readability and overall narrative structure.

Comment-4. “But some of the data sources in India are…”. What is the relevance of this statement? This appears to be somewhat controversial, as the analysis in this study is based on secondary data.

Response: We thank the editor for pointing out this concern. The sentence in question has been reworded to clarify its intent and avoid any potential misunderstanding. The purpose was not to undermine the NFHS dataset used in our analysis, but rather to acknowledge the broader context that certain data sources in India, such as the civil registration system, have incomplete coverage, especially in rural areas.

Comment-5. Which DHS data was used in the manuscript?

Response: We thank to editor for requesting this clarification. The analysis in this manuscript is based on the National Family Health Survey (NFHS-5, 2019–21) dataset (which is India’s version of the Demographic and Health Survey (DHS) program-7).

Comment-6. References are not in the Vancouver style.

Response: Thank you for your valuable comment. We have carefully revised and reformatted all references according to the Vancouver style, adhering to the PLOS ONE journal guidelines.

Comment-7. Some factors which affect the mortality rates were not considered in this study. Mention the limitations regarding the residuals.

Response: We thank the reviewer for this valuable observation. We acknowledge that several socioeconomic and healthcare-related factors influencing child mortality could not be incorporated due to data availability constraints. Furthermore, while the regression residuals were generally small, they do suggest the presence of unexplained variation, which highlights the limitations.

Comment-8. Tables 2 and 3: Mention the 95% CI for the proportion.

Response: We appreciate the reviewer’s suggestion. We have implemented the 95% confidence interval (CI) for the proportions from each models in the revised manuscript.

Comment-9. Table 5: What are X₁ and X₂? Please mention in the footnotes.

Response: We thank the reviewer for pointing this out. We have now added a footnote below Table 5 clarifying the definitions of X1 and X2 as follows:

• X1 = Proportion of dead children among all children born to currently married women aged 15–49 years.

• X2 = Proportion of births of order 4 or higher (4+ birth order).

Review Comments to the Author

Reviewer #1: This study presents an indirect technique for estimating child mortality rates in India using regression-based models. The approach is valuable given the limitations of direct surveys and civil registration systems. However, the manuscript requires improvements in clarity, methodological transparency, and discussion of limitations.

Comment-1: The abstract lacks statistical details on the results. Including R² values or error margins would make it more informative.

Response: We thank the reviewer for this observation. The abstract has been revised to include key statistical measures from our analysis in the revised manuscript.

Comment-2: The introduction highlights the limitations of existing data sources but does not clearly justify why the selected variables (proportion of dead children and 4+ birth order) were chosen over other possible indicators. Please provide a stronger rationale.

Response: We thank the reviewer for this valuable suggestion. In the revised manuscript, we have strengthened the rationale for selecting the two predictor variables. Specifically, we now provide both a theoretical and empirical justification for using the proportion of dead children ever born and proportion of higher-order births (4+).

Furthermore, preliminary correlation analysis using NFHS-5 data demonstrates that both predictors are strongly associated (Pearson’s r > 0.80) with neonatal, infant, and under-five mortality rates, outperforming alternative socioeconomic or healthcare access variables.

Comment-3: The regression analysis approach should be more explicitly described.

Response: We appreciate the reviewer’s suggestion. The Methodology section has been substantially revised to provide a clearer and more detailed description of the regression analysis.

Comment-4: It is unclear how the regression models were validated. Were any statistical goodness-of-fit measures, such as R² or RMSE, used?

Response: We thank the reviewer for this observation. The revised Methodology and Model Validation sections now provide a detailed explanation of the model validation process. Goodness-of-fit was assessed using the coefficient of determination (R2) for each model. Predictive stability was evaluated using Herzberg’s Cross Validity Prediction Power (CVPP) and shrinkage, with smaller shrinkage values indicating greater stability. Practical predictive accuracy was measured by comparing estimated mortality rates with observed NFHS-5 values for each state, applying a ±10% deviation criterion for acceptability.

Comment-5: The manuscript should clarify whether any adjustments (e.g., for confounding variables or biases) were made in the models.

Response: We thank the reviewer for raising this important point. In Methodology section, it explicitly states that no additional adjustments for potential confounding variables or biases were applied beyond the two selected predictors (proportion of dead children and proportion of 4+ birth orders). This choice was deliberate to preserve the simplicity and replicability of the indirect estimation approach, particularly in data-limited contexts.

Comment-6: The manuscript states that data from the National Family Health Survey (2019-21) were used. Were there any data exclusion criteria? How were missing values handled?

Response: We thank the reviewer for this important query. The analysis was conducted using the publicly available data from the National Family Health Survey (2019–21). No exclusion criteria were applied beyond what is already embedded in the NFHS sampling design. With respect to missing values, the dataset used contains very few instances of missing or inconsistent responses for the variables of interest. These cases were automatically excluded from the analysis through listwise deletion, which had negligible impact on the overall sample size and results.

Comment-7: The results indicate a strong alignment between actual and expected rates, but additional statistical evidence (e.g., confidence intervals, p-values) would enhance the credibility of the findings.

Response: We thank the reviewer for this thoughtful suggestion. In the revised manuscript, we have incorporated 95% confidence intervals (CIs) for the proportions obtained from each model to provide stronger statistical support for the findings. In addition, accuracy was assessed by comparing the estimated mortality rates with the observed NFHS-5 values for each state, using a ±10% deviation criterion as the threshold for acceptability.

Comment-8: More comparative analysis with other indirect estimation methods (e.g., Brass method) would strengthen the argument.

Response: We thank the reviewer for this valuable suggestion. In the present study, we have compared our proposed model with the method of Yadava and Tiwari (2003): “An indirect technique for estimations of infant and child mortality: Data analysis from India and Bangladesh” (Health and Population Perspectives and Issues, 26(2), 67–73). This method is conceptually based on the Brass indirect estimation framework, as it employs proportions of dead children to indirectly estimate infant and child mortality in settings with incomplete vital registration. Thus, the comparison already reflects a Brass-derived approach that has been widely applied in the Indian and Bangladeshi context, making it a relevant benchmark for evaluating our model.

Comment-9: The manuscript should discuss the potential policy implications of using this indirect method. A brief comparison with other similar global approaches (e.g., UN Inter-agency Group for Child Mortality Estimation) would be beneficial.

Response: We thank the reviewer for this valuable suggestion. In the revised manuscript, we included in the Discussion section to explicitly address the potential policy implications of the proposed indirect method, particularly for subnational health planning in contexts with incomplete civil registration systems. We have also added a brief comparison with the approach used by the UN Inter-agency Group for Child Mortality Estimation (UN IGME), highlighting similarities in the use of indirect indicators and differences in modeling complexity, scope, and intended application.

Comment-10: The manuscript should explicitly acknowledge limitations, such as assumptions in the regression model, potential biases in NFHS data, and the exclusion of certain socioeconomic factors.

Response: We thank the reviewer for this insightful comment. In the revised manuscript, we have added a dedicated section on “Limitations,” where we discuss assumptions inherent in the regression model, potential biases in NFHS data, and the non-inclusion of certain socioeconomic and healthcare-related factors due to data availability constraints.

Comment-11: Future research directions should be outlined, including how this method could be improved or validated with other datasets.

Response: We thank the reviewer for this suggestion. In the revised manuscript, we have added a “Future Research Directions” paragraph. This addition outlines potential next steps, including validating the proposed method using alternative datasets such as the Sample Registration System (SRS) and District Level Household and Facility Survey (DLHS), incorporating additional socioeconomic and health system predictors, applying longitudinal analyses for model recalibration, and exploring state-specific adjustments to improve accuracy.

Comment-12: Some sentences are unclear or grammatically incorrect. For example:

“But some of the data-source in India is not dependable like civil registration system…” → Consider rewording for clarity:

“Some data sources in India, such as the civil registration system, are unreliable due to incomplete coverage, particularly in rural areas.”

Response: We thank the reviewer for this valuable observation. The sentence has been reworded for clarity and improved grammar in the revised manuscript.

Comment-13: - Proofreading is needed to improve readability.

Response: We appreciate the reviewer’s observation. The entire manuscript has undergone thorough proofreading to improve readability, correct grammatical errors, and enhance clarity in the revised manuscript.

Reviewer #2: Thank you for preparing this important manuscript. I am really unable to appropriately assess the technical parts of this paper because the written English in this manuscript needs significant improvement. I know that English is not the first language of the authors and commend them in putting together this paper; however, in its current form it is very confusing and difficult to understand the writing. I think this paper needs to be edited by someone who speaks fluent English and then resubmitted for review.

There is also no discussion section of the paper which needs to be added.

In addition: there is no such method as "regression line analysis", I am assuming the authors meant a linear regression-if so this needs to be corrected in the entire paper.

Response: We thank the reviewer for these constructive suggestions. In the revised manuscript, we have added a comprehensive Discussion section that interprets our findings in the context of previous studies, outlines policy implications, and addresses limitations.

Regarding the term “regression line analysis”, we agree that this is not standard terminology. Throughout the manuscript, including the Methodology section, we have replaced “regression line analysis” with the appropriate statistical terms “linear line regression technique” (general, covers both simple and multiple regression)”.

Attachment Submitted filename: Response to Reviewers.docx

# Peer review: Decision Letter 1

**DOI:** 10.1371/journal.pone.0353613.r003

**Authors:** Ashish Wasudeo Khobragade

16 Jun 2026

Dear Dr. Singh,

Thank you for submitting your manuscript to PLOS ONE. After careful consideration, we feel that it has merit but does not fully meet PLOS ONE’s publication criteria as it currently stands. Therefore, we invite you to submit a revised version of the manuscript that addresses the points raised during the review process.

Please submit your revised manuscript by Jul 31 2026 11:59PM. If you will need more time than this to complete your revisions, please reply to this message or contact the journal office at plosone@plos.org. When you're ready to submit your revision, log on to https://www.editorialmanager.com/pone/ and select the 'Submissions Needing Revision' folder to locate your manuscript file.

- A letter that responds to each point raised by the academic editor and reviewer(s). You should upload this letter as a separate file labeled 'Response to Reviewers'.
- A marked-up copy of your manuscript that highlights changes made to the original version. You should upload this as a separate file labeled 'Revised Manuscript with Track Changes'.
- An unmarked version of your revised paper without tracked changes. You should upload this as a separate file labeled 'Manuscript'.

If you would like to make changes to your financial disclosure, please include your updated statement in your cover letter. Guidelines for resubmitting your figure files are available below the reviewer comments at the end of this letter.

If applicable, we recommend that you deposit your laboratory protocols in protocols.io to enhance the reproducibility of your results. Protocols.io assigns your protocol its own identifier (DOI) so that it can be cited independently in the future. For instructions see: https://journals.plos.org/plosone/s/submission-guidelines#loc-laboratory-protocols. Additionally, PLOS ONE offers an option for publishing peer-reviewed Lab Protocol articles, which describe protocols hosted on protocols.io. Read more information on sharing protocols at https://plos.org/protocols?utm_medium=editorial-email&utm_source=authorletters&utm_campaign=protocols.

As the corresponding author, your ORCID iD is verified in the submission system and will appear in the published article. PLOS supports the use of ORCID, and we encourage all coauthors to register for an ORCID iD and use it as well. Please encourage your coauthors to verify their ORCID iD within the submission system before final acceptance, as unverified ORCID iDs will not appear in the published article. *Only*  the individual author can complete the verification step; PLOS staff *cannot*  verify ORCID iDs on behalf of authors.

We look forward to receiving your revised manuscript.

Kind regards,

Ashish Wasudeo Khobragade, MD

Academic Editor

PLOS One

Journal Requirements:

1. If the reviewer comments include a recommendation to cite specific previously published works, please review and evaluate these publications to determine whether they are relevant and should be cited. There is no requirement to cite these works unless the editor has indicated otherwise.

2. Please review your reference list to ensure that it is complete and correct. If you have cited papers that have been retracted, please include the rationale for doing so in the manuscript text, or remove these references and replace them with relevant current references. Any changes to the reference list should be mentioned in the rebuttal letter that accompanies your revised manuscript. If you need to cite a retracted article, indicate the article’s retracted status in the References list and also include a citation and full reference for the retraction notice.

[Note: HTML markup is below. Please do not edit.]

Reviewers' comments:

Reviewer's Responses to Questions

**Comments to the Author**

Reviewer #1: All comments have been addressed

Reviewer #3: (No Response)

**********

2. Is the manuscript technically sound, and do the data support the conclusions?

Reviewer #1: Yes

Reviewer #3: Yes

**********

3. Has the statistical analysis been performed appropriately and rigorously?

Reviewer #1: I Don't Know

Reviewer #3: Yes

**********

4. Have the authors made all data underlying the findings in their manuscript fully available?

The PLOS Data policy

Reviewer #1: Yes

Reviewer #3: Yes

**********

5. Is the manuscript presented in an intelligible fashion and written in standard English?

Reviewer #1: Yes

Reviewer #3: Yes

**********

Reviewer #1: The authors have satisfactorily addressed all major comments. The revised manuscript demonstrates improved clarity, methodological transparency, and contextual discussion. I have no further substantive concerns, and I recommend acceptance after minor language or formatting checks.

Reviewer #3: The Editor

PLOS ONE Journal

11 June 2026

Subject: Reviewer Comments on

“Indirect Technique to Estimate Neonatal, Infant, and Under-Five Mortality Rates for India and its States”

(Manuscript ID: PONE-D-25-04899R1)

Dear Editor,

I appreciate the authors’ efforts in addressing an important and relevant topic. The manuscript provides useful insights into the Indirect Technique to Estimate Neonatal, Infant, and Under-Five Mortality Rates for India and its States. However, a few minor revisions are required to improve clarity and methodological transparency.

Reviewer Comments

1. Title:

Please consider including the study design in the title.

2. Abstract:

2.1 It is recommended to add keywords as a subheading.

2.2 In the Methods section, please specify the sample size used in the analysis, including the number of currently married women aged 15–49 years included from the National Family Health Survey (NFHS-5, 2019–21).

3. Methodology (Page 6):

Please provide appropriate references supporting the regression-based mortality estimation method and justify the rationale for selecting the predictor variables used in the analysis.

4. Software:

Please specify the statistical software used, along with its version.

5. Formatting requirement:

Please include the following section before the References, in accordance with journal guidelines:

• List of Abbreviations (in alphabetical order)

Recommendation

Minor Revision

Best regards,

Zainab Mahmood Al-Zadjali

Reviewer

Department of Family Medicine and Public Health

College of Medicine and Health Sciences

Sultan Qaboos University

Muscat, Sultanate of Oman

zainab.alzadjali@yahoo.com

**********

what does this mean?). If published, this will include your full peer review and any attached files.

If you choose “no”, your identity will remain anonymous but your review may still be made public.

**Do you want your identity to be public for this peer review?**  For information about this choice, including consent withdrawal, please see our Privacy Policy

Reviewer #1: **Yes:** Dr. Faham Khamesipour

Reviewer #3: **Yes:** Zainab Mahmood Al-Zadjali

**********

[NOTE: If reviewer comments were submitted as an attachment file, they will be attached to this email and accessible via the submission site. Please log into your account, locate the manuscript record, and check for the action link "View Attachments". If this link does not appear, there are no attachment files.]

To ensure your figures meet our technical requirements, please review our figure guidelines: https://journals.plos.org/plosone/s/figures

You may also use PLOS’s free figure tool, NAAS, to help you prepare publication quality figures: https://journals.plos.org/plosone/s/figures#loc-tools-for-figure-preparation.

NAAS will assess whether your figures meet our technical requirements by comparing each figure against our figure specifications.

Attachment Submitted filename: 11_6_2026_ Under-Five Mortality Rates_ R1 Zainab.docx

# Author comment: Author response to Decision Letter 2

**DOI:** 10.1371/journal.pone.0353613.r004

24 Jun 2026

Reviewer Comments

1. Title:

Please consider including the study design in the title.

Reply: Thank you for this valuable suggestion. We agree that including the study design in the title improves clarity and better reflects the methodological framework of the study. Accordingly, we have revised the title to include the study design as suggested.

2. Abstract:

2.1 It is recommended to add keywords as a subheading.

Reply: Thank you for the helpful suggestion. We agree that including keywords as a separate subheading after the abstract improves the discoverability and indexing of the manuscript. Accordingly, we have added a Keywords section below the abstract in the revised manuscript.

2.2 In the Methods section, please specify the sample size used in the analysis, including the number of currently married women aged 15–49 years included from the National Family Health Survey (NFHS-5, 2019–21).

Reply: Thank you for this valuable suggestion. We agree that explicitly reporting the sample size improves the methodological clarity of the study. Accordingly, we have revised the Methods section to specify the total number of participants included in the analysis, namely the number of currently married women aged 15–49 years selected from the National Family Health Survey (NFHS-5, 2019–21).

3. Methodology (Page 6):

Please provide appropriate references supporting the regression-based mortality estimation method and justify the rationale for selecting the predictor variables used in the analysis.

Reply: The regression-based indirect estimation method used in this study is supported by established demographic literature, including Brass (1975) [Reference 1], which provides the theoretical foundation for indirect mortality estimation using incomplete data, and Yadava and Tiwari (2003) [Reference 12], who successfully applied regression-based techniques for estimating infant and child mortality in India and Bangladesh.

The predictor variables were selected based on both theoretical relevance and empirical evidence. The proportion of dead children among all children born to currently married women aged 15–49 years is a widely accepted indirect indicator of child mortality and has been used in previous demographic estimation studies. In addition, the proportion of births of order 4 or higher (4+ birth order) was included because higher birth order is associated with elevated child mortality risks due to maternal depletion and resource dilution. These predictors also showed strong associations with neonatal, infant, and under-five mortality rates in the present analysis.

4. Software:

Please specify the statistical software used, along with its version.

Reply: Thank you for this valuable suggestion. We have now specified the software used in the revised manuscript. All data management, data analysis, and statistical analyses were performed using Microsoft Excel and Stata Statistical Software, Version 16 (StataCorp LLC, College Station, TX, USA).

5. Formatting requirement:

Please include the following section before the References, in accordance with journal guidelines:

• List of Abbreviations (in alphabetical order)

Reply: Thank you for this helpful suggestion. In accordance with the journal guidelines, we have added a List of Abbreviations section before the References and arranged all abbreviations in alphabetical order.

Attachment Submitted filename: Response to Reviewers (R2).docx

# Peer review: Decision Letter 2

**DOI:** 10.1371/journal.pone.0353613.r005

**Authors:** Ashish Wasudeo Khobragade

27 Jul 2026

Dear Dr. Singh,

Thank you for submitting your manuscript to PLOS ONE. After careful consideration, we feel that it has merit but does not fully meet PLOS ONE’s publication criteria as it currently stands. Therefore, we invite you to submit a revised version of the manuscript that addresses the points raised during the review process.

Please submit your revised manuscript by Sep 10 2026 11:59PM. If you will need more time than this to complete your revisions, please reply to this message or contact the journal office at plosone@plos.org. When you're ready to submit your revision, log on to https://www.editorialmanager.com/pone/ and select the 'Submissions Needing Revision' folder to locate your manuscript file.

- A letter that responds to each point raised by the academic editor and reviewer(s). You should upload this letter as a separate file labeled 'Response to Reviewers'.
- A marked-up copy of your manuscript that highlights changes made to the original version. You should upload this as a separate file labeled 'Revised Manuscript with Track Changes'.
- An unmarked version of your revised paper without tracked changes. You should upload this as a separate file labeled 'Manuscript'.

If you would like to make changes to your financial disclosure, please include your updated statement in your cover letter. Guidelines for resubmitting your figure files are available below the reviewer comments at the end of this letter.

If applicable, we recommend that you deposit your laboratory protocols in protocols.io to enhance the reproducibility of your results. Protocols.io assigns your protocol its own identifier (DOI) so that it can be cited independently in the future. For instructions see: https://journals.plos.org/plosone/s/submission-guidelines#loc-laboratory-protocols. Additionally, PLOS ONE offers an option for publishing peer-reviewed Lab Protocol articles, which describe protocols hosted on protocols.io. Read more information on sharing protocols at https://plos.org/protocols?utm_medium=editorial-email&utm_source=authorletters&utm_campaign=protocols.

As the corresponding author, your ORCID iD is verified in the submission system and will appear in the published article. PLOS supports the use of ORCID, and we encourage all coauthors to register for an ORCID iD and use it as well. Please encourage your coauthors to verify their ORCID iD within the submission system before final acceptance, as unverified ORCID iDs will not appear in the published article. *Only*  the individual author can complete the verification step; PLOS staff *cannot*  verify ORCID iDs on behalf of authors.

We look forward to receiving your revised manuscript.

Kind regards,

Ashish Wasudeo Khobragade, MD

Academic Editor

PLOS One

Journal Requirements:

1. If the reviewer comments include a recommendation to cite specific previously published works, please review and evaluate these publications to determine whether they are relevant and should be cited. There is no requirement to cite these works unless the editor has indicated otherwise.

2. Please review your reference list to ensure that it is complete and correct. If you have cited papers that have been retracted, please include the rationale for doing so in the manuscript text, or remove these references and replace them with relevant current references. Any changes to the reference list should be mentioned in the rebuttal letter that accompanies your revised manuscript. If you need to cite a retracted article, indicate the article’s retracted status in the References list and also include a citation and full reference for the retraction notice.

[Note: HTML markup is below. Please do not edit.]

Reviewers' comments:

Reviewer's Responses to Questions

**Comments to the Author**

Reviewer #3: All comments have been addressed

Reviewer #4: (No Response)

**********

2. Is the manuscript technically sound, and do the data support the conclusions?

Reviewer #3: Yes

Reviewer #4: Yes

**********

3. Has the statistical analysis been performed appropriately and rigorously?

Reviewer #3: Yes

Reviewer #4: Yes

**********

4. Have the authors made all data underlying the findings in their manuscript fully available?

The PLOS Data policy

Reviewer #3: Yes

Reviewer #4: Yes

**********

5. Is the manuscript presented in an intelligible fashion and written in standard English?

Reviewer #3: Yes

Reviewer #4: Yes

**********

Reviewer #3: 29 June 2026

The Editor

PLOS ONE

Subject: Reviewer Comments on "Indirect Technique to Estimate Neonatal, Infant, and Under-Five Mortality Rates for India and its States: A Population-Based Cross-Sectional Study" (Manuscript ID: PONE-D-25-04899R2)

Dear Editor,

I appreciate the authors' efforts in addressing an important and relevant public health topic. The manuscript provides useful insights into the indirect estimation of neonatal, infant, and under-five mortality rates in India and its states using a population-based cross-sectional approach.

After reviewing the revised manuscript and the authors' responses, I am satisfied that the authors have adequately addressed all reviewer comments and concerns raised during the previous review rounds. The revisions have improved the clarity and quality of the manuscript.

Reviewer Comments

1. Title

The authors have addressed the comment regarding the inclusion of the study design in the title. However, I noticed a punctuation/typographical error, with a duplicated colon before “A Population-Based Cross-Sectional Study.”

2. Abstract

The authors have adequately addressed the comments regarding the abstract, except for the inclusion of the sample size in the abstract.

3. Methodology (Page 6)

The authors have provided appropriate references supporting the regression-based mortality estimation method and have justified the rationale for selecting the predictor variables used in the analysis.

4. Software

The authors have specified the statistical software used, including the software version.

5. Formatting Requirements

The authors have addressed the formatting requirements in accordance with the journal guidelines and added an alphabetical list of abbreviations.

Recommendation

Accept.

Best regards,

Zainab Mahmood Al-Zadjali

Reviewer

Department of Family Medicine and Public Health

College of Medicine and Health Sciences

Sultan Qaboos University

Muscat, Sultanate of Oman

zainab.alzadjali@yahoo.com

Reviewer #4: This interesting manuscript has been substantially improved and the authors have addressed most of the comments from the previous reviews. I have a few remaining comments:

i) though the readability is markedly improved, there are a few remaining grammatical errors - I suggest an additional round of proofreading by a fluent English speaker.

ii) R-squared values are provided for all models and offer meaningful information regarding model fit. I suggest adding a sentence regarding interpretation of R-squared for the benefit of readers with limited statistical training.

**********

what does this mean?). If published, this will include your full peer review and any attached files.

If you choose “no”, your identity will remain anonymous but your review may still be made public.

**Do you want your identity to be public for this peer review?**  For information about this choice, including consent withdrawal, please see our Privacy Policy

Reviewer #3: **Yes:**  Zainab Mahmood Dilshad Al-Zadjali

Reviewer #4: No

**********

[NOTE: If reviewer comments were submitted as an attachment file, they will be attached to this email and accessible via the submission site. Please log into your account, locate the manuscript record, and check for the action link "View Attachments". If this link does not appear, there are no attachment files.]

To ensure your figures meet our technical requirements, please review our figure guidelines: https://journals.plos.org/plosone/s/figures

You may also use PLOS’s free figure tool, NAAS, to help you prepare publication quality figures: https://journals.plos.org/plosone/s/figures#loc-tools-for-figure-preparation.

NAAS will assess whether your figures meet our technical requirements by comparing each figure against our figure specifications.

Attachment Submitted filename: 29_6_2026_ Under-Five Mortality Rates_ R2 Zainab.docx

# Author comment: Author response to Decision Letter 3

**DOI:** 10.1371/journal.pone.0353613.r006

29 Jul 2026

We sincerely thank the Academic Editor for the careful evaluation of our manuscript and for providing us with the opportunity to revise it. We are grateful for the valuable comments and suggestions, which have helped us improve the quality and clarity of the manuscript.

Journal Requirements:

1. If the reviewer comments include a recommendation to cite specific previously published works, please review and evaluate these publications to determine whether they are relevant and should be cited. There is no requirement to cite these works unless the editor has indicated otherwise.

Reply: We carefully reviewed the reviewers' comments and confirmed that no specific previously published works were recommended for citation. Therefore, no additional references were added on this basis.

2. Please review your reference list to ensure that it is complete and correct. If you have cited papers that have been retracted, please include the rationale for doing so in the manuscript text, or remove these references and replace them with relevant current references. Any changes to the reference list should be mentioned in the rebuttal letter that accompanies your revised manuscript. If you need to cite a retracted article, indicate the article’s retracted status in the References list and also include a citation and full reference for the retraction notice.

Reply: We carefully reviewed the reference list to ensure that it is complete and accurate. We also verified that none of the cited references have been retracted. Accordingly, no changes related to retracted publications were required.

Reviewers Comment

Reviewer-3

1. Title

Comment 1: The authors have addressed the comment regarding the inclusion of the study design in the title. However, I noticed a punctuation/typographical error, with a duplicated colon before “A Population-Based Cross-Sectional Study.

Reply: Thank you for your observation. We have corrected the punctuation/typographical error by removing the duplicated colon from the title. The title has been revised accordingly.

2. Abstract

Comment 2: The authors have adequately addressed the comments regarding the abstract, except for the inclusion of the sample size in the abstract.

Reply: Thank you for your valuable comment. We have added the study sample size in the Abstract, as suggested. The revised Abstract now includes this information.

3. Methodology (Page 6)

Comment 3: The authors have provided appropriate references supporting the regression-based mortality estimation method and have justified the rationale for selecting the predictor variables used in the analysis.

Reply: We sincerely thank the reviewer for the positive assessment.

4. Software

Comment 4: The authors have specified the statistical software used, including the software version.

Reply: We thank the reviewer for this positive comment.

5. Formatting Requirements

Comment 5: The authors have addressed the formatting requirements in accordance with the journal guidelines and added an alphabetical list of abbreviations.

Reply: We thank the reviewer for this positive comment.

Reviewer-4

This interesting manuscript has been substantially improved and the authors have addressed most of the comments from the previous reviews. I have a few remaining comments:

Comment 1: Though the readability is markedly improved, there are a few remaining grammatical errors - I suggest an additional round of proofreading by a fluent English speaker.

Reply: We sincerely thank the reviewer for this helpful suggestion. The manuscript has undergone an additional round of careful English proofreading, and the remaining grammatical and typographical errors have been corrected throughout the manuscript.

Comment 2: R-squared values are provided for all models and offer meaningful information regarding model fit. I suggest adding a sentence regarding interpretation of R-squared for the benefit of readers with limited statistical training.

Reply: Thank you for your valuable suggestion. We have added a brief sentence explaining the interpretation of the R-squared values to help readers better understand the model fit. The manuscript has been revised accordingly.

Attachment Submitted filename: Response to Reviewers (R3).docx

# Peer review: Decision Letter 3

**DOI:** 10.1371/journal.pone.0353613.r007

**Authors:** Ashish Wasudeo Khobragade

26 Aug 2026

Indirect Technique to Estimate Neonatal, Infant, and Under-Five Mortality Rates for India and its States: : A Population-Based Cross-Sectional Study

PONE-D-25-04899R3

Dear Dr. Singh,

We’re pleased to inform you that your manuscript has been judged scientifically suitable for publication and will be formally accepted for publication once it meets all outstanding technical requirements.

Within one week, you’ll receive an e-mail detailing the required amendments. When these have been addressed, you’ll receive a formal acceptance letter and your manuscript will be scheduled for publication.

An invoice will be generated when your article is formally accepted. Please note, if your institution has a publishing partnership with PLOS and your article meets the relevant criteria, all or part of your publication costs will be covered. Please make sure your user information is up-to-date by logging into Editorial Manager at Editorial Manager® and clicking the ‘Update My Information' link at the top of the page. For questions related to billing, please contact billing support.

If your institution or institutions have a press office, please notify them about your upcoming paper to help maximize its impact. If they’ll be preparing press materials, please inform our press team as soon as possible -- no later than 48 hours after receiving the formal acceptance. Your manuscript will remain under strict press embargo until 2 pm Eastern Time on the date of publication. For more information, please contact onepress@plos.org.

Kind regards,

Ashish Wasudeo Khobragade, MD

Academic Editor

PLOS One

Additional Editor Comments (optional):

Reviewers' comments:

Reviewer's Responses to Questions

**Comments to the Author**

Reviewer #4: All comments have been addressed

**********

2. Is the manuscript technically sound, and do the data support the conclusions?

Reviewer #4: Yes

**********

3. Has the statistical analysis been performed appropriately and rigorously?

Reviewer #4: Yes

**********

4. Have the authors made all data underlying the findings in their manuscript fully available?

The PLOS Data policy

Reviewer #4: Yes

**********

5. Is the manuscript presented in an intelligible fashion and written in standard English?

Reviewer #4: Yes

**********

Reviewer #4: (No Response)

**********

what does this mean?). If published, this will include your full peer review and any attached files.

If you choose “no”, your identity will remain anonymous but your review may still be made public.

**Do you want your identity to be public for this peer review?**  For information about this choice, including consent withdrawal, please see our Privacy Policy

Reviewer #4: No

**********

# Editor report: Acceptance letter

**DOI:** 10.1371/journal.pone.0353613.r008

**Authors:** Ashish Wasudeo Khobragade

PONE-D-25-04899R3

PLOS One

Dear Dr. Singh,

I'm pleased to inform you that your manuscript has been deemed suitable for publication in PLOS One. Congratulations! Your manuscript is now being handed over to our production team.

At this stage, our production department will prepare your paper for publication. This includes ensuring the following:

* All references, tables, and figures are properly cited

* All relevant supporting information is included in the manuscript submission,

* There are no issues that prevent the paper from being properly typeset

You will receive further instructions from the production team, including instructions on how to review your proof when it is ready. Please keep in mind that we are working through a large volume of accepted articles, so please give us a few days to review your paper and let you know the next and final steps.

Lastly, if your institution or institutions have a press office, please let them know about your upcoming paper now to help maximize its impact. If they'll be preparing press materials, please inform our press team within the next 48 hours. Your manuscript will remain under strict press embargo until 2 pm Eastern Time on the date of publication. For more information, please contact onepress@plos.org.

You will receive an invoice from PLOS for your publication fee after your manuscript has reached the completed accept phase. If you receive an email requesting payment before acceptance or for any other service, this may be a phishing scheme. Learn how to identify phishing emails and protect your accounts at https://explore.plos.org/phishing.

If we can help with anything else, please email us at customercare@plos.org.

Thank you for submitting your work to PLOS One and supporting open access.

Kind regards,

PLOS One Editorial Office Staff

on behalf of

Dr. Ashish Wasudeo Khobragade

Academic Editor

PLOS One
